# Evaluation of high resolution ultrasound as a tool for assessing the 3D volume of blood clots during *in vitro* thrombolysis

**DOI:** 10.1038/s41598-017-06089-z

**Published:** 2017-07-24

**Authors:** Laurent Auboire, Jean-Michel Escoffre, Damien Fouan, Jean-René Jacquet, Frédéric Ossant, Jean-Marc Grégoire, Ayache Bouakaz

**Affiliations:** 1 UMR Inserm U930, Université François-Rabelais de Tours, Tours, France; 2 CHRU de Tours; INSERM CIC 1415, 37000 Tours, France

## Abstract

Thrombosis is a major cause of several diseases, *i.e*. myocardial infarction, cerebral stroke and pulmonary embolism. Thrombolytic therapies are required to induce fast and efficient recanalization of occluded vessels. To evaluate the *in vitro* efficacy of these thrombolytic strategies, measuring clot dissolution is essential. This study aimed to evaluate and validate high resolution ultrasound as a tool to assess the exact volume of clots in 3D and in real time during *in vitro* thrombolytic drug testing. This new method was validated by measuring the effects of concentration range of recombinant tissue type plasminogen activator on a blood clot during complete occlusion or 70% stenosis of a vessel. This study shows that high resolution ultrasound imaging allows for a real-time assessment of the 3D volume of a blood clot with negligible inter- and intra-operator variabilities. The conclusions drawn from this study demonstrate the promising potential of high resolution ultrasound imaging for the *in vitro* assessment of new thrombolytic drugs.

## Introduction

Thrombosis is a major cause of several cardiovascular diseases; among them, the deadliest are myocardial infarction and acute cerebral stroke. Indeed, ischemic heart disease and stroke are the first and second causes of death worldwide, with 7.4 million and 6.7 million in 2012, respectively^1^. Blood clots are formed by platelets, white and red blood cells, and a polymerized fibrin network^2^. The interruption of bloodstream by a blood clot leads to tissue hypoxia and necrosis if the bloodstream is not quickly restored. During ischemic stroke, 1.9 million neurons are lost each minute and the ischemic brain ages 3.6 years each hour if no treatment is administered^3^. The time before recanalization of occluded vessels is the most important predictive factor of a bad outcome.

Nowadays, recombinant tissue type plasminogen activator (Rt-PA) is the only clinically-approved therapy for ischemic stroke. As an enzyme, this drug catalyzes the conversion of plasminogen to plasmin, the major enzyme responsible for the cleavage of fibrin fibers, which leads to clot breakdown. The early use of Rt-PA in clinical applications revealed that its therapeutic effectiveness is strongly limited by potentially adverse side effects such as hemorrhages and its narrow time window (4.5 h)^4^. Although treatment of ischemic stroke using Rt-PA as a single agent has shown effective recanalization of an artery in only 42% of patients, these rates can reach 85% when used in combination with stent retrieval, leading to a more important percentage of patients who are functionally independent at 90 days^5^. This last method, which consists of removing the blood clot with an intra-arterial (i.a.) catheter, is mainly used when Rt-PA is contra-indicated. However, the number of patients eligible for i.a. therapy is limited because of the availability of facilities and the diameter of the artery (<2 mm)^5^. To overcome these limitations, the development of more efficient and targeted thrombolytic drugs is required to dissolve blood clots as quickly as possible while minimizing side effects, including hemorrhages.

The *in vitro* evaluation of new thrombolytic therapies is a major step toward pre-clinical evaluation and validation. At this stage, a blood clot is inserted in a flow circuit to identify the mechanisms of action of thrombolytic agents as well as to evaluate their efficacy in terms of dissolving clots in an *in vitro* controlled environment. The *in vitro* efficacy of new thrombolytic therapies is assessed by measuring the speed of clot breakdown using different methods; the advantages and limitations of these methods are summarized in Table 1.

**Table 1:** The main methods used to measure clot dissolution during *in vitro* thrombolysis.

Methods	Principle	Non-contact	Lysis kinetics	Price	Main limits
Weight^17^	Weight of the clot before and after treatment	No	No	+++	Influenced by dehydration;
					No volume measurement
Imaging^18^	Taking an image of one side of the clot	Yes	Yes	++	2D evaluation;
					Approximate volume
Optical coherence tomography^12^	Light waves are reflected at different depths within the sample	Yes	Yes	—	Low light penetration
					Radiolabeled
D-Dimer dosage^19^	Radiolabeled iodine is integrated into the fibrin molecule and liberated during thrombolysis	Yes	Yes	—	No volume measurement;
					radioactive component
Hydrodynamic parameter^10^	Difference in pressure before and after clot dissolution	Yes	Yes	+	Requires flow
Turbidity of the solution^20^	Thrombolysis liberates particles, thereby increasing the turbidity of the solution	+/−	Yes	+	Not possible with flow
High resolution ultrasound imaging^Present study^	3D ultrasound imaging of blood clots	No	Yes	+	Requires the presence of red blood cells in the clot

Among the available imaging modalities, ultrasound imaging is a powerful technique both in preclinical research and clinical applications. This imaging modality combines many advantages, including real-time imaging, non-invasiveness, high spatial and temporal resolution, the absence of ionizing irradiation, portability and a low cost. Ultrasound imaging provides anatomical (*e.g*., detection, localization), morphological (*e.g*., dimensions, volume) and functional (*e.g*., blood perfusion, expression of endothelial markers) information. In this context, the aim of this study was to investigate whether high frequency ultrasound imaging is an efficient method for the real-time assessment of the *in vitro* efficacy of a thrombolytic drug. Hence, we investigated the monitoring potential of ultrasound imaging to evaluate the therapeutic efficacy of a range of concentrations of Rt-PA on blood clots inserted in a flow circuit, mimicking two clinical relevant situations^6^. The first one was total occlusion of one of the two branches of a bifurcation, thus simulating complete occlusion of an artery, which causes 90% of all ischemic strokes. The second one was partial occlusion of the same branch, thus mimicking arterial stenosis, responsible for 10% of all ischemic strokes. This methodology addressed the following research questions: (i) Is high frequency ultrasound imaging a reproducible method to measure the 3D volume of a blood clot? (ii) Can high resolution ultrasound imaging be used to assess the *in vitro* therapeutic efficacy of Rt-PA?

## Material and Methods

All methods were carried out in accordance with relevant French guidelines and regulations.

### Preparation of human blood clots

Human blood samples were collected from healthy volunteers using an approved clinical research protocol. Informed consent of volunteers was obtained (French Blood Service, Tours, France). Blood was anticoagulated with 3.2% sodium citrate (Becton, Dickinson and Company, Le Pont de Claix, France). Clotting was initiated by the addition of calcium chloride (500 mM), which acts as a co-factor in the coagulation process by counteracting sodium citrate. The blood (80 μL) was placed in a custom polyolefin carrier (1.2 mm length, 1.6 mm inside diameter, 1.85 mm outside diameter). Polyolefin is highly biocompatible, and thus does not interfere with protein activity^7^. In addition, polyolefin shows low ultrasound attenuation, thus allowing high frequency ultrasound imaging of the blood clot. This method preserved the physiological coagulation process without adding a foreign body into the clot and allowed for performing serial tests without manipulating the sample.

The blood was allowed to clot (*i.e*., complete coagulation and fibrin network retraction) at 37 °C for 24 hours (Incucell, MMM Medcenter Einrichtungen GmbH, München, Germany). The carrier was left horizontally overnight in a Petri dish containing 0.9% saline serum (to avoid dehydration) to induce 70% stenosis (*i.e*., clot retraction left a lumen), while complete occlusion was achieved by orienting the carrier vertically in a 1.5 mL Eppendorf tube (Fischer Scientific SAS, Illkirch, France) filled with recalcified blood.

### Flow system

**Figure 1:**
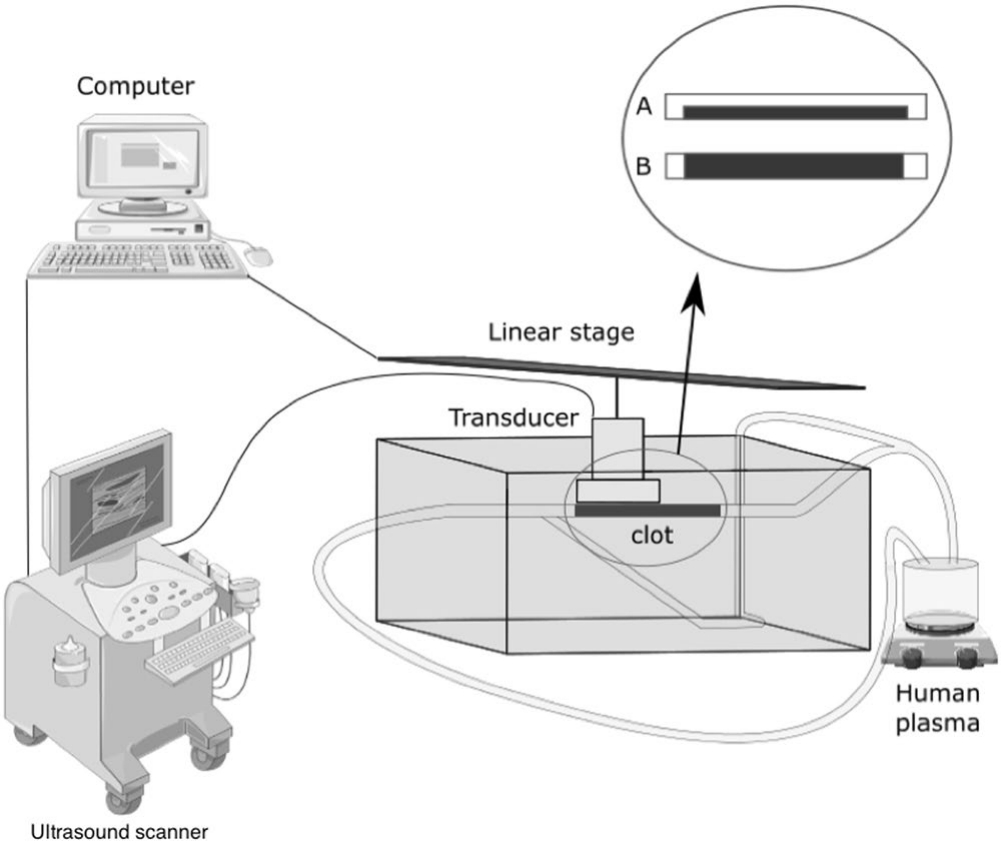
Schematic of *in vitro* setup for the thrombolysis experiments. Drawing of ultrasound scanner, computer and human plasma, have been obtained from Servier Medical Art of Servier under the Creative Commons Attribution 3.0 France license.

The flow system simulated the hemodynamic conditions of the middle cerebral artery (Fig. 1). This system was an open circuit consisting of 1.6 mm (internal diameter) flexible silicone tubing. One end was connected to reservoir filled with 15 mL of recalcified human plasma (French Blood Service, Tours, France). The tube system had a bifurcation to two arms, which were submerged in a 37 °C water bath. One of the arms was occluded with a human blood clot. A programmable hotplate magnetic stirrer (RH basic 2, IKA^®^ Werke GmbH & Co. KG, Staufen, Germany) maintained the plasma temperature at 37 °C. A peristaltic pump (MCP Process IP65, Cole-Parmer GmbH, Wertheim, Germany) provided constant flow at 20 mL/min to the system, mimicking the shear stress of the cerebral arterial circulation (24.4 Dynes/cm^2^ in the main branch).

### High frequency ultrasound imaging

A Vevo^®^ 2100 ultrasound scanner (VisualSonics Inc., Toronto, Canada) with an MS-550D probe (22–55 MHz; 40 µm axial and 90 µm lateral resolutions) and a LIMES^®^ 150 linear stage (OWIS, Staufen, Germany) was used to capture 3D images of the blood clot (Fig. 1). The scanning of the whole blood clot was performed in 15 seconds (*i.e*., 1 image/200 µm, 60 images in total), every 5 minutes for 60 minutes. After image acquisition, 3D reconstruction of the blood clot was carried out using Horos^®^ software (GNU Lesser General Public License, Version 3.0), Freecad^®^ (GNU Lesser General Public License, Version 2 or superior) and the clot volume was determined.

### Repeatability and inter-operator reproducibility in the 3D blood clot volume

The repeatability and the inter-operator variability (two operators, LA and JME) of clot volume assessments using high frequency ultrasound imaging were investigated on ten individual and serial measurements of the clot volume mimicking either 70% stenosis or full occlusion. All experiments were performed in recalcified plasma to mimic clinical conditions. In addition, to keep the volume of the clot stable, the pump was stopped to avoid mechanical volume variation during the experiment.

### *In vitro* Rt-PA delivery

Rt-PA (Activase^®^, Genentech, San Francisco, CA) was reconstituted and frozen after being split into 100 µL aliquots. Rt-PA was thawed prior to each experiment and diluted with recalcified human plasma to obtain the final concentrations of 0.3 µg/ml, 3 µg/ml (*i.e*., the human steady state concentration in stroke thrombolysis^8^ and 30 µg/ml). The diluted Rt-PA solution was delivered at a flow rate of 20 mL/min for 60 min.

A total of 16 blood clots mimicking 70% stenosis (Fig. 1A) were divided into four experimental groups: (1) control group (*i.e*., w/o pharmacological treatment), (2) Rt-PA × 0.1 (*i.e*., 0.3 µg/ml Rt-PA), (3) Rt-PA group (*i.e*., 3 µg/ml Rt-PA) and (4) Rt-PA × 10 group (*i.e*., 30 µg/ml Rt-PA). Then, a total of 12 blood clots simulating full occlusion (Fig. 1B) was divided into three experimental groups: (1) control group (*i.e*., w/o pharmacological treatment), (2) Rt-PA group (*i.e*., 3 µg/ml Rt-PA) and (3) Rt-PA × 10 group (*i.e*., 30 µg/ml Rt-PA).

### Measurement of surface exchange between the clot and the plasma

Surface exchange between the clot and the plasma was determined as follows: (1) for the full occlusion setup, we measured the area of the clot on the first image; (2) for the stenosis setup, the areas of the clot on the first and the last images of the clot were measured and the areas on each slice in contact with the plasma were summed, (thickness of one slice: 200 µm.

### Statistical analysis

Descriptive statistics were performed using Xlstat^®^ software (Addinsoft; Paris, France). Statistical analysis of the results was carried out using the non-parametric Kruskal-Wallis test and Dunn’s multiple comparison post-test. The repeatability and the inter-operator variability were assessed using the variation coefficient and the Bland-Altman test, respectively. For comparison of the surface exposed to plasma, the Mann-Whiney test was used. Significance was defined as *p* < 0.05 (NS, non-significance, ^*^ *p* < 0.05, ^**^ *p* < 0.01 and ^***^ *p* < 0.001).

## Results

### Assessing 3D blood clot volume using high resolution ultrasound imaging: repeatability and inter-operator reproducibility

**Figure 2:**
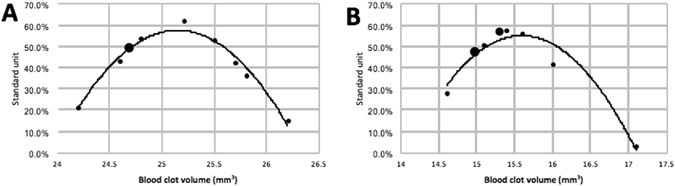
Normal distribution of repeated measures for stenosis (**A**) and for full occlusion (**B**). The small dot represents one measure. The larger dot represents two measures (giving the same value).

As shown in the Table 2, the mean clot volumes for the stenosis and full occlusion conditions were 15.44 ± 0.69 mm^3^ and 25.14 ± 0.63 mm^3^, respectively. The Shapiro-Wilk normality test revealed that these results followed a normal distribution for both situations (stenosis setup: W = 0.8714, *p* = 0.1039; full occlusion setup: W = 0.95, *p* = 0.669) (Fig. 2). The repeatability of the measurements was assessed using the variation coefficient. This coefficient was 2.54% for stenosis and 4.49% for full occlusion. These results reveal that our imaging modality allowed for repeated measurements of clot volume.

**Table 2:** Intra-operator repeatability of the 3D clot volume measurement.

	Full occlusion clot	Stenosis clot
Mean clot volume (mm^3^)	25.14	15.44
Standard deviation (SD)	0.63	0.69
Variation coefficient	2.54%	4.49%

**Figure 3:**
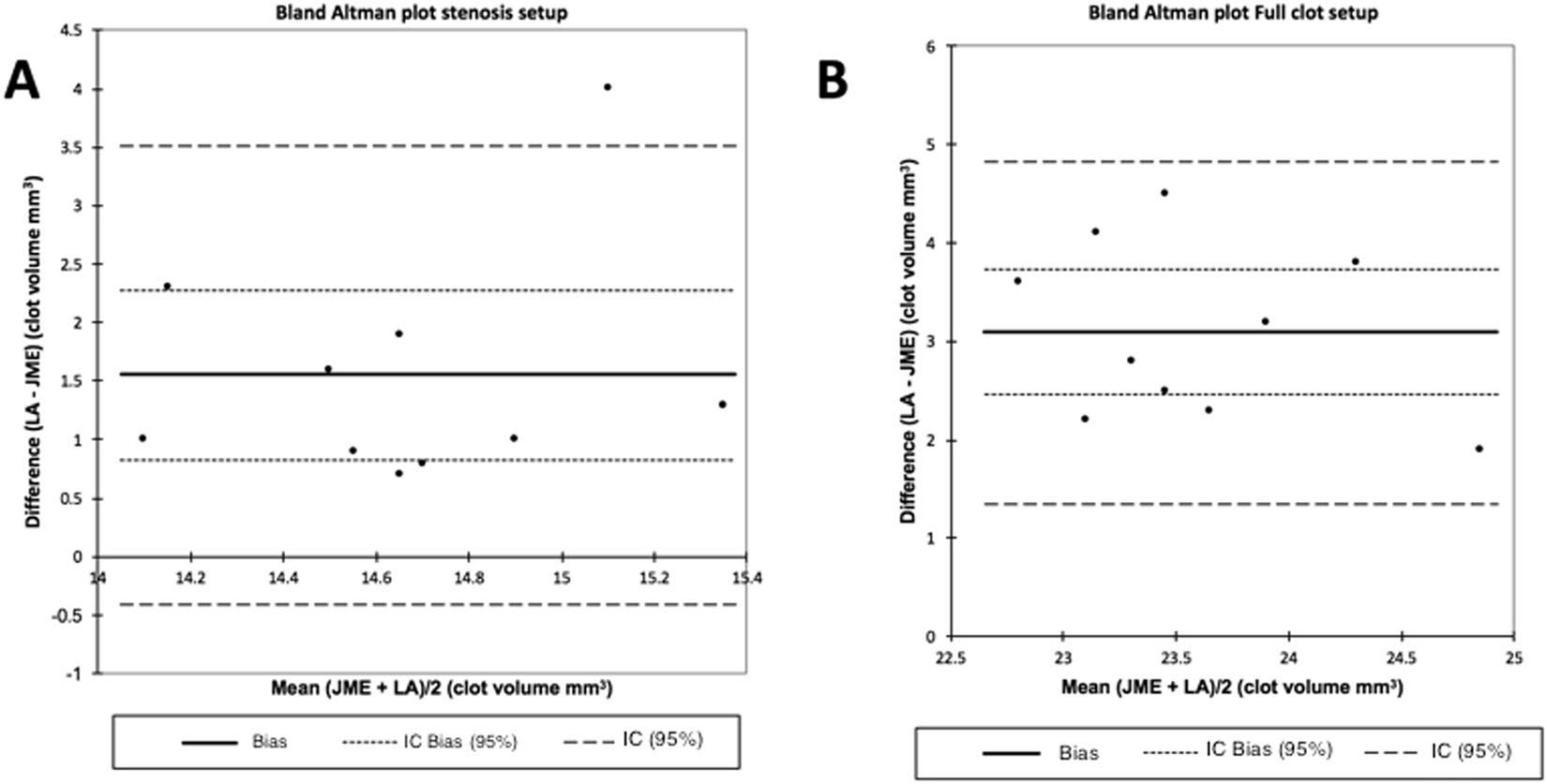
Bland-Altman plot for stenosis (**A**) and the occlusion (**B**) describing the inter-operator reproducibility.

The inter-operator reproducibility was assessed using the Bland-Altman plot (Fig. 3). The measured bias for the 70% stenosis and the full occlusion setups were 1.55 ± 1.00 mm^3^ and 3.09 ± 0.88 mm^3^, respectively. The results suggest that high frequency ultrasound imaging provides a reliable and reproducible assessment of the blood clot volume. Based on these results, this imaging modality was chosen to monitor the thrombolytic efficacy of Rt-PA in the *in vitro* stenosis and full occlusion models.

### Thrombolytic efficacy evaluation: *in vitro* delivery of Rt-PA in the stenosis model

**Figure 4:**
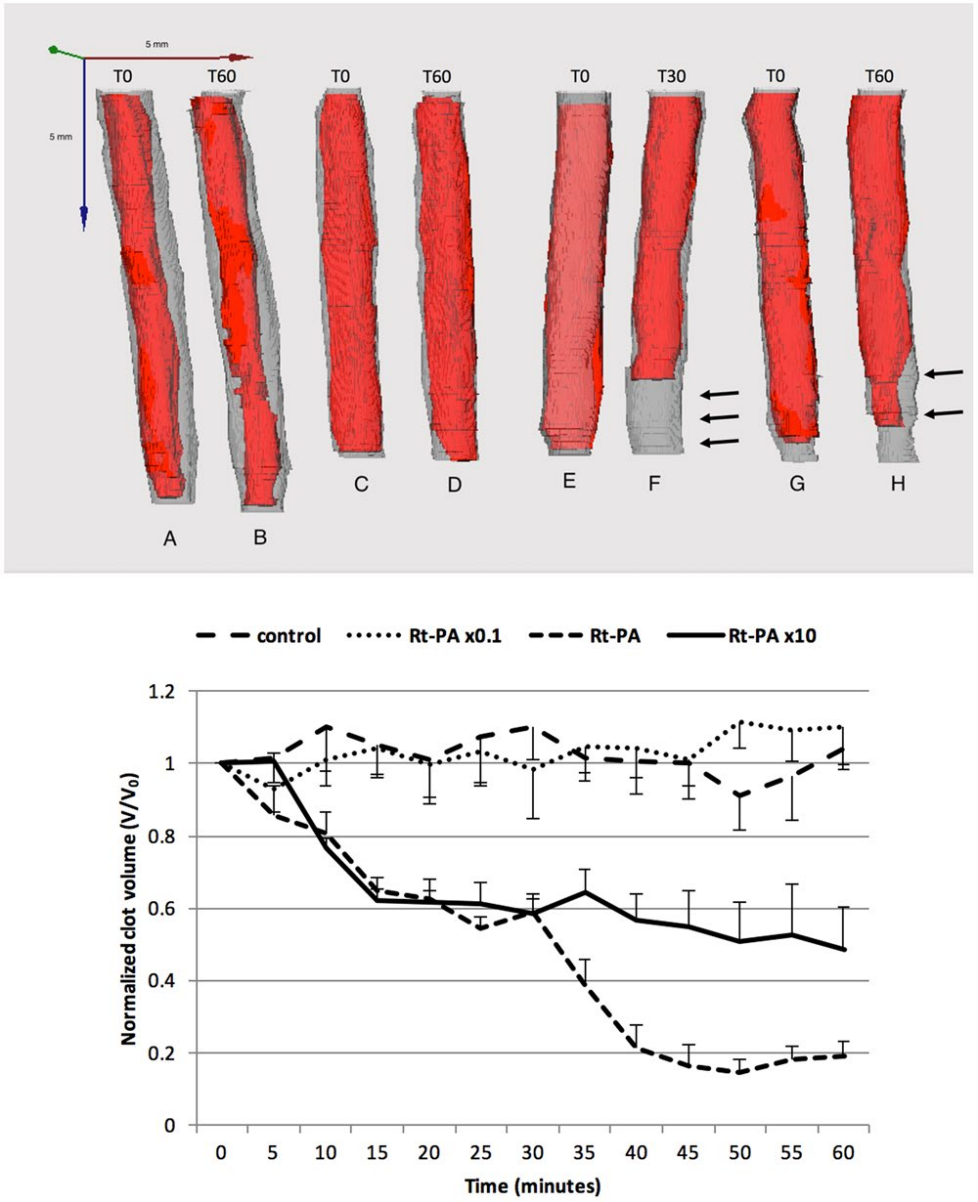
Thrombolytic efficacy of *in vitro* after Rt-PA delivery in the stenosis model. Blood clots were treated with a range of Rt-PA alone in a range of concentrations (0, 0.3, 3, 30 µg/ml). The blood clot volume was assessed every 5 min for 60 min using high frequency ultrasound imaging. 3D reconstruction of clots was performed at T0 and T60 (Top), except for the Rt-PA condition (T30) as the clot was almost lysed at T60. A, B Rt-PA x0,1; C-D: control; E-F: Rt-PA; G-H: Rt-PAx10 (Arrows indicating the lysis of the clot). Video 1 is showing the clot volume at T0 and T30 in the Rt-PA condition. Data expressed as mean ± SEM were calculated from four independent experiments (Bottom).

The thrombolytic efficacy of a range of Rt-PA concentrations on the *in vitro* stenosis model was assessed by high frequency ultrasound imaging; the results are shown in Fig. 4. The figure displays in the top panel 3D ultrasound images of reconstructed blood clots and in the lower panel the ultrasound measured clot volume for different Rt-PA concentrations. The exposure of the blood clot to circulating plasma without Rt-PA induced a slight change in the clot volume over time (60 min), with no significant difference in comparison to the clot volume at the beginning of the experiment (0 min; *p* > 0.05).

As shown in Fig. 4, when the blood clots were treated with the low concentration of Rt-PA alone (*i.e*., 0.3 µg/ml), the clot volume was not significantly different from the control conditions without Rt-PA (*p > 0.05). At the Rt-PA concentration of 3 µg/ml (*i.e*., human steady state concentration in stroke thrombolysis^8^), the clot volume decreased significantly compared to the low concentration of Rt-PA (*p* < 0.05) and the control condition (*p* < 0.05). The clot volume reached 2.36 ± 3.51 mm^3^ after 60 min treatment with Rt-PA treatment in the stenosis setup (Fig. 4), thus leading to an 81% decrease of clot volume.

Blood clots treated with the high Rt-PA concentration of 30 µg/ml showed a significant decrease in volume in comparison to the low concentration of Rt-PA (*p* < 0.05) and the control condition (*p* < 0.05) (Fig. 4). For the first 30 minutes, this treatment caused a similar decrease in clot volume compared to the Rt-PA concentration of 3 µg/ml (*p* > 0.05) (Fig. 3). Surprisingly, the high Rt-PA concentration (30 µg/ml) significantly impaired clot breakdown, with a decrease of 52% of clot volume after 60 min, in comparison to the Rt-PA concentration of 3 µg/ml (*p* < 0.05) (Fig. 4). These results clearly show that the thrombolytic efficacy of Rt-PA depends on the administered Rt-PA concentration, with a minimum threshold efficacy found between 0.3 and 3 µg/ml.

### *In vitro* delivery of Rt-PA in the full occlusion model

Blood clots mimicking a stroke (*i.e*., complete occlusion model; Fig. 1B) were treated with either 3 µg/ml or 30 µg/ml of Rt-PA and compared to the control condition (*i.e*., circulating plasma without Rt-PA). As previously described, the thrombolytic efficacy of Rt-PA was evaluated by assessing the blood clot volume using high frequency ultrasound imaging.

**Figure 5:**
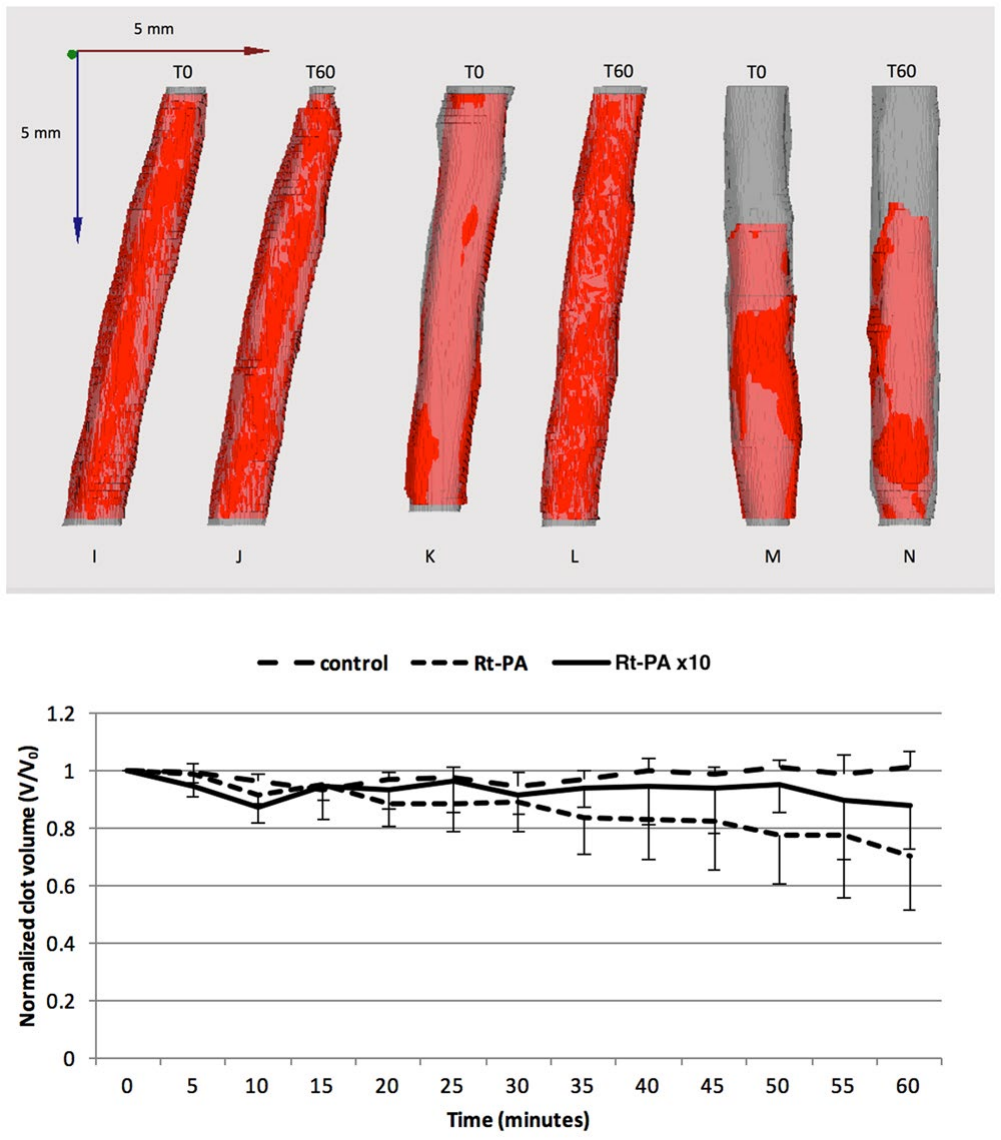
*In vitro* Rt-PA delivery in the full occlusion model. Blood clots were treated with a range of Rt-PA alone in a range of concentrations (0, 3, 30 µg/ml). The blood clot volume was assessed every 5 min for 60 min using high frequency ultrasound imaging. 3D reconstructions of clots were made at T0 and T60 (top). I-J: Control; K-L: Rt-PA; M-N: Rt-PAx10. Data expressed as mean ± SEM were calculated from four independent experiments (bottom).

As shown in Fig. 5, the exposure of blood clots to human plasma without Rt-PA did not result in significant changes in the clot volume based on ultrasound images (*i.e*., 60 min; *p* > 0.05). Surprisingly, when blood clots were treated with the Rt-PA concentration of 3 µg/ml, the clot volume was not significantly different from the control condition without Rt-PA (*p* > 0.05) (Fig. 5). In addition, the high Rt-PA concentration of 30 µg/ml did not induce a significant change in clot volume in comparison to the low concentration of Rt-PA (*p* > 0.05) and the control condition (*p* > 0.05) (Fig. 5).

**Figure 6:**
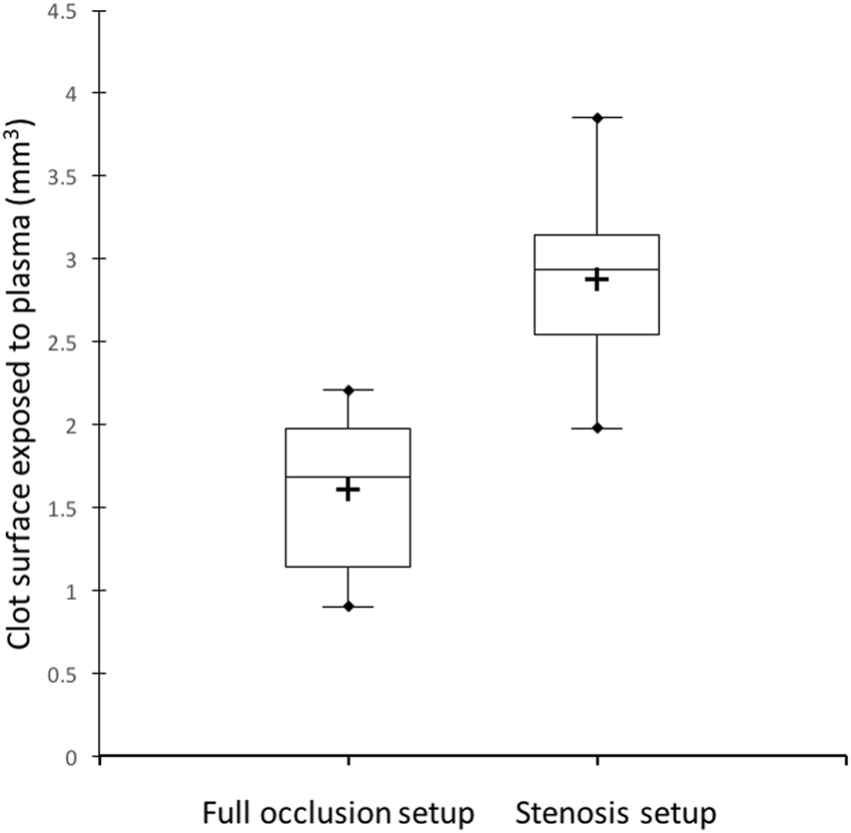
Surface contact between the blood clot and the plasma in the stenosis and full occlusion models.

These results obtained with high resolution ultrasound imaging suggest that the *in vitro* thrombolytic efficacy of Rt-PA in the stroke model is dependent of the existence of flow and the degree of exchange surface between the blood clot and the plasma. Indeed, this exchange surface was significantly higher in the stenosis model compared to the full occlusion model (*p* < 0.0001) (Fig. 6).

## Discussion

The present study examined the monitoring potential of high frequency ultrasound imaging for a real-time assessment of the *in vitro* efficacy of a thrombolytic drug. First, we characterized this imaging modality *in vitro*, showing that high frequency ultrasound imaging is a reproducible and a reliable method to measure the blood clot volume in *in vitro* stenosis and stroke models (Figs 2 and 3). Unlike in previous studies^9^, the clot volume was stable for 60 minutes, with a flow system (without Rt-PA delivery) mimicking the shear stress of the cerebral arterial circulation (24.4 Dynes/cm^2^ in the main branch).

As mentioned earlier, high frequency ultrasound imaging combines many advantages including real-time, non-destructive imaging, with high spatial and temporal resolution, the absence of ionizing irradiation and a low cost. Recently, Roessler *et al*. developed a hydrodynamic approach to assess the thrombolytic efficacy of drugs^10^. This method provides time-continuous measurements compared to ultrasound imaging where the clot scan requires 15 seconds. However, the main limitation of the hydrodynamic approach is that it requires circulating flow, which does not represent the most common clinical situation in stroke^6, 11^. Moreover, Kim *et al*. reported their assessment of the *in vitro* thrombolytic efficacy of drugs using high resolution optical coherence tomography (OCT)^12^. This method enables high resolution imaging of the fibrin network as this network is fully transparent to ultrasound. However, OCT does not provide in-depth information of the blood clot because of attenuation due to red blood cells, whereas ultrasound imaging explores the full structure of the clot. Although human blood clots are composed of variable proportions of fibrin, red and white blood cells in acute stroke^2^, the use of clots generated from platelet-rich plasma (*i.e*., without red blood cells) has also been reported^9^. However, high frequency ultrasound imaging fails to image these platelet-enriched clots (data not shown), because there is no backscattered echo provided by aggregated red blood cells^13^. Consequently, this imaging modality cannot monitor the thrombolytic efficacy of treatments based on a hemolytic effect, as already reported with sonothrombolysis^14^. Altogether, these data suggest that the combination of high frequency ultrasound imaging and OCT could be a promising strategy to investigate *in vitro* thrombolytic efficacy.

Subsequently, we demonstrated that high frequency ultrasound imaging is a relevant method to monitor the thrombolytic effect of concentration range of Rt-PA in the *in vitro* stenosis and full occlusion models (Figs 4 and 5). Based on the results collected from this *in vitro* study, we confirmed that the action of Rt-PA at the surface is limited when the plasma flow is interrupted by the presence of full clot occlusion (Fig. 6). Indeed, Rt-PA forms a ternary complex with plasminogen and fibrin fibers. In this conformation, the affinity constant of Rt-PA for plasminogen is 1 μM in the presence of fibrin fibers; this constant increases to 33 μM in the absence of fibrin fibers^15^. This ternary complex catalyzes the conversion of plasminogen to plasmin, the major enzyme responsible for the cleavage of fibrin fibers, which leads to clot breakdown. Hence, a limited surface area for Rt-PA to act upon decreases the ability to form this ternary complex and impairs the thrombolytic efficacy of the Rt-PA. In addition, the presence of plasma flow might impose shear stress on the blood clot, thus accelerating its dissolution.

Our validation study showed that a high dose of Rt-PA (*i.e*., 30 µg/ml) failed to induce efficient thrombolysis compared to lower doses (0.3 or 3 µg/ml). This phenomenon is known as *“plasminogen steal”*. Plasmin is quickly inactivated by plasma protease inhibitors, such as α-2-antiplasmin (half-life of plasmin in human plasma is 0.1 second). A high dose of Rt-PA catalyzes the conversion of plasminogen, located both in the plasma and inside the blood clot, into plasmin. This mass-produced plasmin might not interact sufficiently with fibrin fibers due to competition for binding site in fibrin fiber. Unlike to normal dose of Rt-PA (3 µg/ml), all the plasminogen is consumed and no more plasmin is produce at high dose of Rt-PA (30 µg/ml), leading to an impairment of thrombolysis^16^.

A detrimental side-effect of thrombolysis is the division of the clot in two or more clot fragments that may occlude smaller vessels downstream, and cause stroke. For instance, this case may occur in brain’s small vessels. This technology is interesting, as its real-time capability may help decipher the kinetics of thrombolysis, *i.e.*, the reduction of blood clot volume as a function of time, and detect a potential fractioning of the initial blood clot that is at risk for the vasculature downstream. Figure 4 is interesting, since such a time profile of clot volume reduction may indicate an abrupt decrease in blood clot volume, if this clot would have lost a major piece that would then be at risk downstream. This aspect is be interesting to understand the mode of action of the drug on blood clots, and help design drug formulations that would ensure the non-fractioning of these clots, and the dissolution of the outer layers with a slower kinetics profile.

## Conclusion

In summary, the present work suggests that high frequency ultrasound imaging is a reliable imaging modality to assess the *in vitro* thrombolytic efficacy of drugs. The experimental validation of this method showed that the thrombolytic efficacy of Rt-PA is related to the presence of flow around the blood clot and that high concentrations of this drug impair its thrombolytic efficacy. High frequency ultrasound imaging may be a new method to investigate the therapeutic efficacy of thrombolytic drugs. Moreover, this imaging modality should be combined with other ultrasound imaging modes such as shear wave elastography and photoacoustic imaging for a complete evaluation of the fibrinolysis process through the acquisition and analysis of multi-parametric modifications during lysis.

## Electronic supplementary material

Supplementary Information

Video 1
