# Supplementary material for: Evaluation of high resolution ultrasound as a tool for assessing the 3D volume of blood clots during in vitro thrombolysis
Source: Sci Rep. 2017 Jul 24;7:6211. doi: 10.1038/s41598-017-06089-z (PMC5524902; doi:10.1038/s41598-017-06089-z)
Supplement: Supplementary file 1 — Supplementary Information [file 41598_2017_6089_MOESM1_ESM.pdf]

Evaluation of high resolution ultrasound as a tool for assessing the 3D volume of blood clots during *in vitro* thrombolysis

Laurent Auboire[1], Jean-Michel Escoffre[1], Damien Fouan[1], Jean-René Jacquet[1], Frédéric Ossant[1, 2], Jean-Marc Grégoire[1], Ayache Bouakaz[1*]

[1]UMR Inserm U930, Université François-Rabelais de Tours, France.

[2]CHRU de Tours, CIC-IT, 37044 Tours cedex, France.

Corresponding author: A. Bouakaz, Ph.D., UMR Inserm U930 Imagerie et Cerveau, Université François-Rabelais. 10 bd Tonnellé, 37032 Tours Cedex 1, France. Tel: +33(0)247366142. Email address: ayache.bouakaz@univ-tours.fr

Video 1: 3D Reconstruction of clot volume at T0 and T30 in the Rt-PA (3µg/ml) condition in the stenosis setup.
